# Inorganic Phosphate as “Bioenergetic Messenger” Triggers M2‐Type Macrophage Polarization

**DOI:** 10.1002/advs.202306062

**Published:** 2024-01-21

**Authors:** Xiaoqing Sun, Zhiyu Li, Xiang Wang, Jing He, Yao Wu

**Affiliations:** ^1^ National Engineering Research Center for Biomaterials Sichuan University Chengdu Sichuan 610064 P. R. China

**Keywords:** AMPK‐mTOR axis, CaP scaffold, energy metabolism, macrophage polarization, phosphorous

## Abstract

The effects of calcium phosphate (CaP) materials on macrophage polarization state vary with their physicochemical properties. The study aims to elucidate the impact of phosphate ion‐mediated energy metabolism on M2 macrophage polarization and the corresponding regulatory mechanism. The phosphate ions released from CaP ceramic as bioenergetic factor is identified; its concentration is closely associated with the polarized state. After being taken up by the sodium‐dependent phosphate transporter 1, extracellular phosphate ions produce energy via oxidative phosphorylation by facilitating tricarboxylic acid flux, thereby contributing to M2 macrophage polarization. Further mechanistic analysis reveals that the elevation of the bioenergetic basis can drive macrophage M2 polarization via the AMP‐activated protein kinase‐mammalian target of rapamycin (AMPK‐mTOR) axis. Another regulatory effect is that of the adenosine triphosphate (ATP), a signaling molecule. Intracellular ATP is released into the extracellular space and degraded to adenosine, which serves as a signaling molecule through the A2b adenosine receptor to activate the cyclic adenosine monophosphate (cAMP) pathway, thereby promoting M2 macrophage polarization. Overall, these findings may transform the existing knowledge on cell metabolism and energy homeostasis from bystanders to pivotal factors guiding M2 macrophage polarization and have implications for the future design of biomimetic CaP scaffolds.

## Graphical abstract

Phosphate ions released from CaP ceramics, after being taken up by SLC20a1, produce energy via OXPHOS by facilitating TCA flux, thereby promoting M2 macrophage polarization. This is likely due to increased energy availability, which activates mTOR signaling by decreasing AMPK activity. Moreover, ATP serves as an extracellular autocrine and paracrine signaling molecule that promotes M2 macrophage polarization via A2b adenosine receptor.

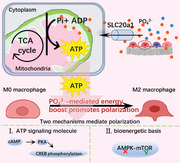

## 1 Introduction

The immune microenvironment of bone regeneration sites is crucial for reconstructing the tissue regeneration process. A substantial body of evidence supports the pivotal role of macrophages in various wound‐healing processes. Additionally, it has been shown that an advanced paradigm for regulating macrophages is essential for achieving successful bone regeneration.^[^ ^1^, ^2^, ^3^ ^]^ Biomaterials‐based bone substitutes are widely used for bone tissue regeneration in large bone defects. Our study, along with others, has shown that biomaterials incorporating calcium phosphate (CaP), a key inorganic component of natural bone tissue, can promote bone tissue formation in vivo.^[^ ^4^, ^5^, ^6^ ^]^ However, the effectiveness of these materials in repairing bone defects is closely linked to the response and polarization states of macrophages. Therefore, to effectively utilize CaP‐based materials for bone defect repair, it is crucial to understand both the osteoimmunological status and molecular mechanisms involved in macrophage polarization within a CaP mineral environment.

CaP biomaterials usually tend to degrade calcium and phosphate ions (i.e., Ca^2+^ and PO_4_^3−^), forming a microenvironment capable of regulating bone regeneration.^[^ ^7^ ^]^ Among the various biomaterials containing CaP, those that easily dissociate into Ca^2+^ and PO_4_^3−^ ions have been found to enhance bone healing. This observation is further supported by research indicating that exposing osteoblasts and progenitor cells to a medium rich in either Ca^2+^ or PO_4_^3−^ promotes osteogenic differentiation.^[^ ^8^, ^9^, ^10^ ^]^ Despite numerous studies highlighting the potential impact of CaP minerals, Ca^2+^, and PO_4_^3−^, on osteogenic performance, a majority of molecular investigations have primarily focused on the regulatory role of Ca^2+^. For example, Wen et al. have reported that an influx of extracellular Ca^2+^ via L‐type calcium channels would accelerate osteogenic differentiation of osteoprogenitor cells and bone formation.^[^ ^11^ ^]^ Additionally, PO_4_^3−^ has a critical, but often overlooked, role in supporting osteogenesis. In addition to apatite formation, studies have shown that PO_4_^3−^ is an important driver of osteogenic processes caused by an increase in cellular energy metabolism.^[^ ^9^, ^12^ ^]^ Extracellular PO_4_^3−^ are transported by sodium‐dependent phosphate transporter 1 (SLC20a1, or PiT‐1), a sodium‐phosphate symporter that transports PO_4_^3−^ ions from the extracellular milieu into the cytoplasm and participates in the tricarboxylic acid (TCA) cycle and oxidative phosphorylation (OXPHOS), resulting in elevated intracellular adenosine triphosphate (ATP) levels.^[^ ^13^, ^14^ ^]^ The expansion of CaP matrices to investigate the impact of PO_4_^3−^ on osteogenesis has revealed that an elevation in intracellular ATP levels is advantageous for initiating a signaling cascade that prompts MSC differentiation into the osteoblastic lineage in response to CaP.^[^ ^15^, ^16^ ^]^ Additionally, Liu et al. and Bossche et al., have substantiated that ATP release is an efficacious approach for regulating energy demand during the initial phase of tissue repair.^[^ ^17^, ^18^ ^]^

Recent advancements have underscored the pivotal significance of energy requirements in the activation state and related functions of macrophages.^[^ ^19^ ^]^ Cell transformation frequently involves changes in energy metabolism that facilitate adaptation to cellular functions and reprogramming interactions within the cellular environment.^[^ ^20^, ^21^ ^]^ M1 macrophages exhibit metabolic reprogramming toward glycolysis, whereas M2 macrophages rely on OXPHOS for energy production (i.e., ATP). Consequently, aerobic glycolysis and OXPHOS play distinct roles in regulating the macrophage phenotype and function.^[^ ^22^ ^]^ Despite PO_4_^3−^ having shown positive enhancement in boosting osteogenic differentiation via modulating intracellular energy metabolism,^[^ ^23^, ^24^ ^]^ the role of PO_4_^3−^ in modulating the metabolic programming of macrophages is unclear. Considering the positive role of PO_4_^3‐^ in OXPHOS, PO_4_^3−^ ions from the extracellular milieu may provide biochemical and bioenergetic basis for M2 macrophage polarization. Therefore, it is imperative to investigate the impact of PO_4_^3−^ on macrophage polarization, as well as the connection between the role of extracellular PO_4_^3−^ in cell metabolism, final polarization fate selection, and the corresponding molecular mechanisms.

Here, we identified this central link in which the CaP mediated‐rich PO_4_^3−^ environment, taken up through SLC20a1, targeted the energy metabolism to shape the transition of macrophages into M2 subsets, an effect referred to as “metabolic regulation”. Our study showed that extracellular PO_4_^3−^ was critical to promote polarization of macrophages toward regenerative M2 macrophages, by producing energy in the form of ATP through the TCA cycle and OXPHOS. Increased energy availability resulted in the activation of the mammalian target of rapamycin (mTOR) signaling by a decrease in AMP‐activated protein kinase (AMPK) activity, and subsequently switched on M2 macrophage polarization. Moreover, ATP, secreting into the extracellular environment and metabolizing into adenosine, served as extracellular autocrine and paracrine signaling molecules to promote the polarization of M2 macrophages via A2b adenosine receptor. Overall, this study presents a mechanism to demonstrate the beneficial role of phosphate ions in macrophage functional phenotypes and ultimately inspires the development of novel CaP biomaterial designs toward metabolic strategies.

## 2 Results and Discussion

### 2.1 Phosphate Degradability of CaP Ceramics

**Figure 1:**
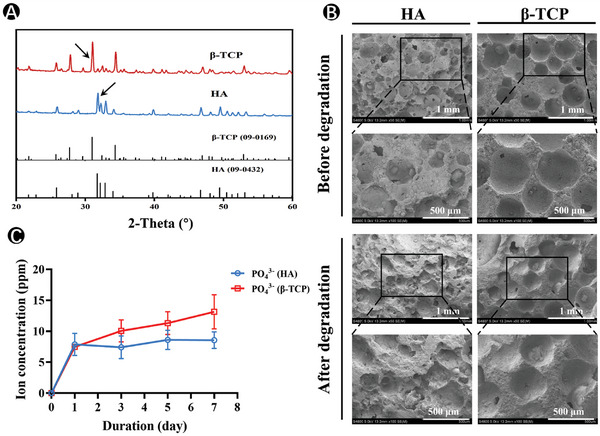
Characterization of CaP ceramics. A) XRD graph of HA and β‐TCP scaffolds. B) SEM morphologies of HA and β‐TCP scaffolds before and after 7‐day immersion in Tris‐HCl solution. C) Phosphate ionic concentrations released from HA and β‐TCP scaffolds (*n* = 3).

Two CaP ceramics (hydroxyapatite (HA) and β‐tricalcium phosphate (β‐TCP)), with porosity of ≈70% with microporous structures are shown in **Figure** **1**. The energy dispersive spectroscopy (EDS) analysis confirmed the corresponding Ca/P ratio for HA and β‐TCP (Figure S1, Supporting Information). X‐ray diffraction (XRD) analysis provided further confirmation that they were indeed HA and β‐TCP, as evidenced by the specific diffraction peaks observed at 2θ = 31.81° (HA) and 2θ = 31.07° (β‐TCP) (Figure 1A). The surface morphologies of the CaP ceramics were examined before and after immersion. After 7 days of immersion in Tris‐HCl buffer solution (pH 7.4), the scaffold topography changed considerably to varying degrees (Figure 1B). Furthermore, the abilities of the HA and β‐TCP ceramics to release PO_4_^3−^ in the Tris‐HCl buffer solution were evaluated (Figure 1C). The PO_4_^3−^ released from β‐TCP ceramic was significantly higher than that from HA ceramic. The concentrations of PO_4_^3−^ continued to increase with incubation time, reaching 15.1 ppm at day 7 in β‐TCP ceramics. Combining the results of surface change and PO_4_^3−^ releasing abilities, it could be concluded that β‐TCP ceramic had stronger degradation abilities than HA ceramic, which was consistent with previous reports.^[^ ^7^ ^]^ Aside to the physical cues provided, it was important to consider the dissolution kinetics and the ability of CaP ceramics to modulate the extracellular mineral environment through dissolution/formation processes, as these factors significantly influenced the biological functions of CaP materials, which have often been overlooked. Our results indicated that the β‐TCP ceramic exhibited a stronger ability to create a CaP‐rich environment than HA ceramic, with more and faster PO_4_^3−^ ions released.

### 2.2 CaP Ceramics Enhance M2 Macrophage Polarization Through SLC20a1

**Figure 2:**
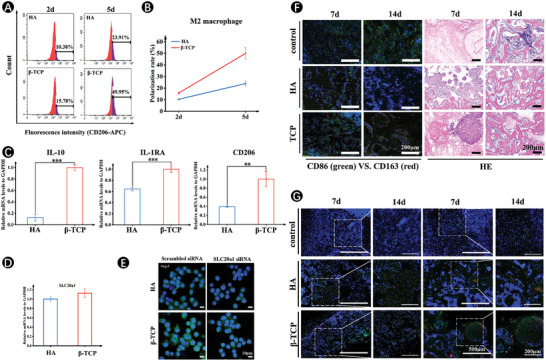
CaP ceramics enhance M2 macrophage polarization through SLC20a1 in vitro and in vivo. A) Flow cytometry analysis of CD206 (M2 marker) in macrophages treated with HA and β‐TCP scaffolds after 2 days and 5 days of culturing. B) Polarization trend of M2‐type macrophages on HA and β‐TCP scaffolds. C,D) Gene expressions of IL‐10, IL‐1RA, CD206 (C), and SLC20a1 (D) after 5 days of culture on HA and β‐TCP scaffolds. E) Immuno‐Fluorescence staining for M2 phenotype marker‐Arg‐1 with SLC20a1 siRNA and corresponding scrambled siRNA after 5 days of culture. F) Immuno‐Fluorescence staining for M2 marker‐CD163 (red fluorescence), M1 marker‐CD86 (green fluorescence) and HE staining at 7 and 14 days in vivo. G) Immuno‐Fluorescence staining for M2 marker‐CD163 (red fluorescence) and SLC20a1 (green fluorescence) at 7 and 14 days in vivo. (*n* = 5. The values presented are the mean ± SD. Statistical significance is assessed by the student's *t*‐test. ^*^ *p* < 0.05, ^**^ *p* < 0.01, and ^***^ *p* < 0.001 vs HA).

We then sought to uncover the role of phosphate ions released from the CaP ceramics in macrophage polarization toward the M2 subtype. The rate of M2 polarization was determined using flow cytometry (**Figure** **2**A,B). The percentage of M2 polarization gradually increased with the increase of incubation time, indicating a shift in macrophage polarization toward the M2 subtype. Moreover, there was a significantly higher proportion of M2 polarization in the β‐TCP group, with high PO_4_^3−^‐rich environment, compared with that in the HA group. At 5 days, the percentage of CD206^+^ cells in the β‐TCP group wase as high as 50%, with the ratio being ∼ 2‐fold greater for HA group, indicating that β‐TCP scaffolds outcompeted HA scaffolds for modulating the polarization of macrophages to M2 phenotype (Figure 2B). Meanwhile, the M2‐polarized‐related parameters, including IL‐10, IL‐1RA, and CD206, as detected by real‐time quantitative reverse transcription polymerase chain reaction (qRT‐PCR), were up‐regulated in the β‐TCP scaffolds, further ascertaining the effectiveness of β‐TCP scaffolds in inducing M2 macrophage polarization (Figure 2C). In addition to M2‐like markers, RAW264.7 cells cultured on β‐TCP scaffolds presented up‐regulation of sodium‐phosphate symporter SLC20a1 expression at 5 days (Figure 2D). When incubation time was extended to 10 days, the expressions of SLC20a1 in RAW264.7 cells treated with β‐TCP scaffold were significantly higher than those in the HA scaffold (Figure S2, Supporting Information). Notably, knockdown of SLC20a1 expression with siRNA (≈50% reduction) led to the decreased immunofluorescent staining for arginase‐1 (Arg‐1), suggesting that the increase in degraded PO_4_^3−^ content from scaffolds may be related to the upregulation of M2 polarization (Figure 2E; Figure S3, Supporting Information).

Our in vivo data also provided an affirmation of the in vitro results. M2 macrophage marker, CD163, was also significantly increased in the bone tissue of β‐TCP scaffold‐treated rats compared with those in HA‐treated rats, especially for 14 days. Many studies have shown that promoting macrophage polarization to the reparative M2 phenotype was important during the eradication of inflammation and tissue regeneration because it can tune an inflammatory microenvironment toward a pro‐regenerative niche.^[^ ^25^, ^26^ ^]^ Correspondingly, HE staining demonstrated that β‐TCP scaffolds with high expression of an M2 macrophage marker showed promising outcomes in fracture repair models (Figure 2F). More importantly, a noteworthy association was identified between the densities of SLC20a1‐positive and CD163‐positive cells. The SLC20a1‐positive cells were dispersed among the CD163‐positive cells. Additionally, a substantial correlation was observed between a higher proportion of SLC20a1‐positive cells and a higher proportion of CD163‐positive cells, thereby reinforcing the significance of the sodium‐phosphate symporter SLC20a1 expression in macrophage polarization (Figure 2G).

### 2.3 Inorganic Phosphate‐Regulated Macrophage Polarization uses SLC20a1

**Figure 3:**
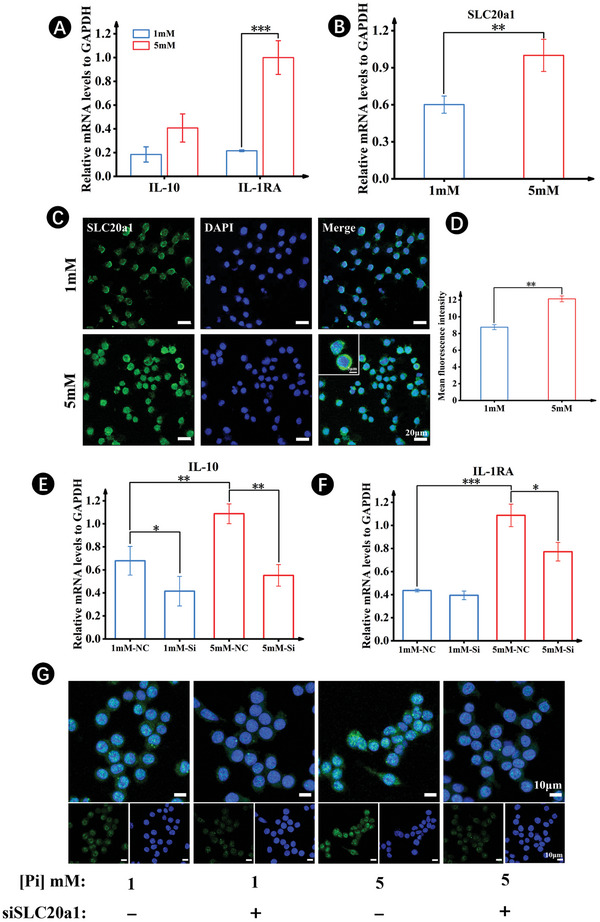
Inorganic phosphate‐regulated macrophage polarization uses SLC20a1. A,B) Gene expressions of IL‐10, IL‐1RA (A), and SLC20a1 (B) after 5 days of culture in growth medium with low (1 mm) and high (5 mm)‐phosphate concentrations. C) Immuno‐Fluorescence staining for SLC20a1. D) Semi‐quantitative analysis of immunofluorescent signals of SLC20a1. E,F) Gene expressions of IL‐10 and IL‐1RA after 5 days of culture in low‐ and high‐phosphate medium with SLC20a1 siRNA and corresponding scrambled siRNA. G) Immuno‐Fluorescence staining for Arg‐1 with SLC20a1 siRNA and corresponding scrambled siRNA after 5 days of culture in low‐ and high‐phosphate medium. (*n* = 5. The values presented are the mean ± SD. Statistical significance is assessed by student's *t*‐test or by one‐way ANOVA with Tukey's post hoc test. ^*^ *p* < 0.05, ^**^ *p* < 0.01, and ^***^ *p* < 0.001).

As the CaP ceramics contributed to the extracellular PO_4_^3−^ and SLC20a1 transported phosphate, further verification of the role of extracellular PO_4_^3−^ on macrophage polarization of RAW264.7 was conducted by culturing the cells in different concentrations of phosphate‐containing medium. Similar to the above‐mentioned results, the RAW264.7 cells cultured in high phosphate (5 mm) medium displayed significantly higher expressions of various M2 markers, including IL‐10 and IL‐1RA, relative to those cultured in low phosphate (1 mm) medium (**Figure** **3**A). In parallel, RAW264.7 cells cultured in high phosphate (5 mm) medium showed higher gene expressions of the sodium‐phosphate symporter SLC20a1 (Figure 3B). This finding was further confirmed by immunofluorescent staining, which demonstrated clear accumulation of SLC20a1 fluorescent signals within the cells (Figure 3C,D). Akin to CaP ceramics, the knockdown of SLC20a1 with siRNA annulled the phosphate‐mediated macrophage polarization of RAW264.7, as evidenced by the downregulation of IL‐10 and IL‐1RA expressions quantified by qRT‐PCR and a greater decrease in fluorescent intensity (Figure 3E–G).

Overall, the finding that M2 macrophage polarization via CaP ceramics and high PO_4_^3−^ medium can be reversed by knocking down the SLC20a1 gene demonstrated that PO_4_^3−^ content in the extracellular environment and its transportation through SLC20a1 were critical mediators of CaP ceramic‐induced M2 polarization in macrophages.

### 2.4 Increase of Intracellular ATP Through the Actions of the TCA and Oxidative Phosphorylation, Which Is Dependent on SLC20a1

**Figure 4:**
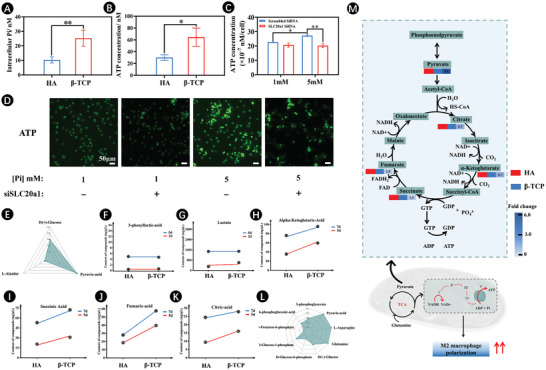
Increase of intracellular ATP through the actions of the TCA and oxidative phosphorylation which is dependent upon SLC20a1. A) Content of intracellular PO_4_^3−^ ion after 5 days of culture on HA and β‐TCP scaffolds. B) Concentration of intracellular ATP after 5 days of culture on HA and β‐TCP scaffolds. C) Concentration of intracellular ATP after 5 days of culture in low‐ and high‐phosphate medium with SLC20a1 siRNA and corresponding scrambled siRNA. D) intracellular ATP staining with quinacrine after 5 days of culture in low‐ and high‐phosphate medium with or without SLC20a1 knockdown. E) A radar chart of metabolite abundance via targeted energy metabolite analysis. F–K) Content of 3‐phenyllactic‐acid (F), lactate (G), alpha‐ketoglutaric‐acid (H), succinic acid (I), fumaric‐acid (J), and citric‐acid (K). L) The radar plot to display the effect of β‐TCP scaffolds on the metabolite abundance. M) Schematic showing the changes in targeted key metabolites involved in TCA cycle. The red and blue represent the HA and β‐TCP scaffolds, respectively. Blue from light to deep indicates the more obvious difference in metabolite for β‐TCP scaffold compared to HA scaffold. (*n* = 3. The values presented are the mean ± SD. Statistical significance is assessed by student's *t*‐test or by one‐way ANOVA with Tukey's post hoc test. ^*^ *p* < 0.05 and ^**^ *p* < 0.01).

One fundamental question remains, however, as to how extracellular PO_4_^3−^influences the transformation of macrophages into M2‐type macrophages. We observed a significant increase in intracellular PO_4_^3−^ of macrophages cultured on β‐TCP scaffolds with high PO_4_^3−^ ion release compared with those on HA scaffolds as measured by the phosphate assay (**Figure** **4**A). Considering that intracellular inorganic phosphate was an essential constituent of ATP, ATP production was examined. As evidenced by a luminescent assay, there was a notable increase in intracellular ATP levels when RAW264.7 cells were cultured on β‐TCP scaffolds for 5 days (Figure 4B). To further confirm whether PO_4_^3−^ was directly involved in ATP synthesis, we cultured macrophages in medium containing 5 mm PO_4_^3−^. Similar to cells grown on CaP ceramics, a significant increase was noted in intracellular ATP in the cells cultured in high PO_4_^3−^ (5 mm) medium compared with that in the cells cultured in low PO_4_^3−^ (1 mm) medium; however, this increase was attenuated upon knockdown of SLC20a1 with siRNA (Figure 4C). This finding was corroborated by the quinacrine staining for intracellular ATP. The signal intensity was weak in low PO_4_^3−^ (1 mm) group, indicating low ATP levels. Conversely, the signal intensity was greatly enhanced in high PO_4_^3−^ (5 mm) group, and this enhancement was reduced upon SLC20a1 knockdown (Figure 4D).

Cellular uptake of phosphate ions represented a key prerequisite as a response to the degradation of CaP ceramics, resulting in changes in the metabolic state. Next, to monitor the impact of the increased intracellular inorganic phosphate on cellular energy metabolism, we tested the abundances of the key metabolites in the macrophages with the stimulation of β‐TCP scaffolds, and HA scaffolds were selected as control. Figure 4E shows a radar chart highlighting the outstanding effects of pyruvic acid on energy metabolism. Pyruvic acid plays a central role in energy metabolism and could be regarded as an important node for the switch between aerobic and anaerobic metabolism.^[^ ^27^ ^]^ Pyruvic acid would be reduced to lactate in the cytoplasm or be oxidized as acetyl coenzyme A in the mitochondria, entering the TCA cycle.^[^ ^28^ ^]^ To identify the pathway by which pyruvic acid was used to produce energy, we first measured the levels of lactate and 3‐phenyllactic‐acid (Figure 4F,G; Table S1, Supporting Information). No noticeable difference in the abundance of lactic acid and 3‐phenyllactic‐acid was found between HA samples and β‐TCP samples. Therefore, we hypothesized that pyruvic acid may be implicated in the mitochondrial TCA cycle to regulate energy metabolism. Of note, key intermediate products in the TCA cycle, including alpha‐ketoglutaric‐acid, succinic acid, fumaric acid, and citric acid were significantly elevated when the cells were cultured on β‐TCP ceramic (Figure 4H–K; Table S1, Supporting Information). This suggested an enhancement of TCA cycle by β‐TCP scaffolds while decreasing the flux toward lactate. It was perhaps relevant that intramitochondrial phosphate increased. Varghese et al. have been confirmed that cells may use extracellular phosphate to increase intramitochondrial phosphate, which could serve as the primary substrate for ATP synthesis in the electron transport chain of mitochondria.^[^ ^9^ ^]^ Increasing mitochondrial membrane potential can boost TCA cycles. Notably, the TCA cycle is a critical event in OXPHOS, and is a more efficient method for ATP generation.^[^ ^29^ ^]^ Metabolic adaptation that occurs during macrophage activation is an essential aspect of macrophage polarization and is instrumental in their function in inflammation.^[^ ^30^, ^31^ ^]^ Several lines of evidence have suggested that M2 macrophages underwent a metabolic shift from the less energy‐efficient glycolytic pathway to the TCA cycle to meet the increasing demand for attenuating inflammation and promoting tissue regeneration.^[^ ^32^, ^33^ ^]^ Here, highly activated energy production, via TCA, enabled β‐TCP scaffold to induce macrophage polarization into M2 phenotypes. We also found that the increase in pyruvic acid was accompanied by the upregulation of asparagine and glutamine abundances (Figure 4L). M2‐type macrophages are more dependent on glutamine influx into the TCA cycle than M1 macrophages.^[^ ^34^, ^35^ ^]^ Asparagine and glutamine can be used to replenish the intermediates in the TCA cycle after deamination.^[^ ^36^ ^]^

Overall, inorganic phosphate exhibited a dose‐dependent effect on ATP accumulation and TCA flux, potentially serving as the underlying mechanism through which it influenced macrophage polarization. β‐TCP ceramics created a CaP‐rich environment that was conducive to the energy requirements necessary for M2 macrophage polarization, achieved through a metabolic shift toward the TCA cycle for ATP production. This stimulation of the TCA cycle not only facilitated the generation of bioenergy essential for macrophage polarization but also yielded metabolites that influenced the phenotype and functionality of macrophages (Figure 4M).

### 2.5 Intracellular ATP Directly Modulates Macrophage Polarization via AMPK‐mTOR Signaling Pathway

**Figure 5:**
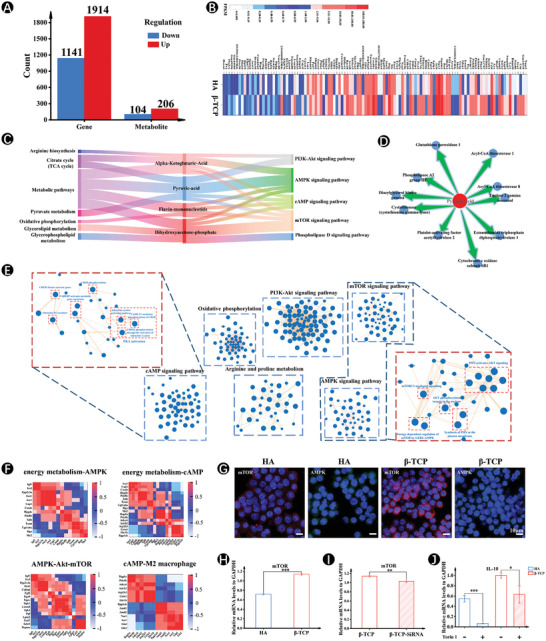
Bioinformatic analysis of overall gene and metabolite expression changes on HA and β‐TCP scaffolds. A) The up‐and‐down‐regulation of gene and metabolite. B) Heat map illustrating the common changed substances in genes and metabolites from HA and β‐TCP scaffolds. C) The Sankey diagram links metabolic pathways and transcriptional pathways through metabolic substances. D) Pyruvic acid is associated with a variety of energy metabolites. E) Subnetworks of the notable enrichment maps created from the selected pathways. F) The Pearson's correlation analysis. The correlation coefficient ranged from −1 to 1 (blue to red) depending on the color intensity. G) Immuno‐Fluorescence staining for mTOR and AMPK after 5 days of culture on HA and β‐TCP scaffolds. H) Gene expressions of mTOR after 5 days of culture on HA and β‐TCP scaffolds. I) Gene expressions of mTOR after 5 days of culture on β‐TCP scaffolds with or without SLC20a1 knockdown. J) Gene expressions of IL‐10 after 5 days of culture on β‐TCP scaffolds with or without Torin1. (*n* = 5. The values presented are the mean ± SD. Statistical significance is assessed by student's *t*‐test. ^*^ *p* < 0.05, ^**^ *p* < 0.01, and ^***^ *p* < 0.001).

Next, the dual‐omics associative analysis, including genomics and non‐targeted metabolomics analysis was employed to elucidate the molecular mechanism by which increased metabolic levels promoted macrophage polarization. A total of 11 268 transcripts and 673 metabolites were quantified in the transcriptome and metabolome, respectively. In comparison to the HA group, the transcriptome of the β‐TCP group revealed the presence of 3055 differentially expressed genes, with 1914 being upregulated and 1141 being downregulated. Additionally, the metabolome analysis identified a total of 310 differentially expressed metabolites, with 206 being upregulated and 104 being downregulated (**Figure** **5**A). Out of these 116 were significantly changed at both the transcriptome and metabolome levels, indicating the coordination of transcriptome and metabolome remodeling, as shown in the heatmap (Figure 5B; Table S2, Supporting Information).

In addition, the conjoint Kyoto Encyclopedia of Gene and Genomes (KEGG) pathway enrichment analysis was conducted to examine the transcriptome and metabolome data. This analysis revealed the emergence of common genes and corresponding metabolites in both genomic and metabolic pathways (Figure 5C). Notably, macrophage activation necessitated metabolic support, thereby requiring polarizing signals to energize and regulate the metabolic pathways. As expected, arginine biosynthesis was also observed. M2 macrophage polarization was characterized, in part, by the ability to metabolize L‐arginine.^[^ ^37^ ^]^ Arginine biosynthesis was associated with PI3K‐Akt signaling pathway, AMPK signaling pathway, cyclic adenosine monophosphate (cAMP) signaling pathway, and mTOR signaling pathway through alpha‐ketoglutaric‐acid, indicating that macrophage polarization was related to the above signaling pathways in β‐TCP‐treated cells. Moreover, TCA cycle and oxidative phosphorylation that were associated with M2‐polarized metabolism state were linked with AMPK and mTOR signaling pathways, indicating that AMPK‐mTOR axis may be involved in β‐TCP‐mediated bioenergy to promote macrophage polarization. In addition, we found indications of the existence of additional regulatory mechanisms. Pyruvate, an important precursor metabolite involved in energy production, was implicated in cAMP signaling pathway, in addition to AMPK‐mTOR axis (Figure 5D).

Furthermore, we sought to establish the interaction networks involved in predicting alterations in cellular signaling cascades using the GeneMANIA. Notably, an interactive network was established between arginine and proline metabolism and oxidative phosphorylation. Numerous genes were associated with oxidative phosphorylation through relatively few downstream pathways, often converging on the AMPK‐mTOR axis and cAMP signaling (Figure 5E).

AMPK is an important energy receptor in cells and decreased energy availability results in AMPK activation and restoration of energy homeostasis. When energy is sufficient, mTOR signaling is activated by a decrease in AMPK activity.^[^ ^38^, ^39^ ^]^ Further PPI analysis using GeneMANIA analysis showed the category of “energy‐dependent regulation mTOR by LKB1‐AMPK”. Specially, the β‐TCP scaffolds increased the synthesis of PIPs at the plasma membrane and PLD activity, leading to greater AKT activation. Activated AKT then phosphorylated mTOR and activated its downstream signaling pathway to promote the polarization of macrophages toward the M2 phenotypes. This finding aligned with previous reports highlighting the importance of AMPK‐mTOR pathway in modulating macrophage polarization toward the M2‐phenotype.^[^ ^40^, ^41^ ^]^

Subsequently, the Pearson's correlation analysis provided additional evidence supporting the significance of the AMPK‐mTOR signaling pathway in β‐TCP‐mediated macrophage polarization. Of particular interest were three regions in the confusion matrix (heatmap) that displayed strong correlation coefficients (red or blue). 1) The energy metabolism was significantly correlated with the AMPK signaling pathway. 2) mTOR was modulated by both AKT and AMPK, which were to regulate mTOR positively and negatively, respectively (Figure 5F; Table S3–S8, Supporting Information).

Finally, the role of AMPK‐mTOR pathway in β‐TCP‐mediated‐energetic level promoting M2 polarization was further confirmed. Immunofluorescence staining and qRT‐PCR results demonstrated that cells cultured on β‐TCP scaffolds exhibited a significant increase in mTOR expressions (Figure 5G,H) and a simultaneous decrease in AMPK intensity, compared with HA scaffolds (Figure 5G). Conversely, mTOR intensity was reduced, following blocking with siSLC20a1 (Figure 5I; Figure S4, Supporting Information). Furthermore, the pharmaceutical inhibition of mTOR signaling by Torin1 attenuated the polarization of macrophages into the M2 phenotype, as evidenced by the downregulated gene expressions of IL‐10; this confirmed that inorganic phosphate increased energy metabolism in macrophages via AMPK‐mTOR signaling pathway, promoting M2 macrophage polarization (Figure 5J).

Overall, elevations in the intracellular PO_4_^3−^ concentration in the β‐TCP scaffolds induced the increased bioenergy level, resulting in AMPK turning off. This was accompanied by phosphorylation modification via AKT, resulting in the release of its inhibitory effect on AMPK and the upregulation of mTOR activity.^[^ ^42^ ^]^ The influence of AMPK‐mTOR‐mediated sensing of environmental and metabolic cues on macrophage polarization was expected, given that macrophage polarization was regulated not only by cytokines and growth factors but also by environmental cues, including nutrients, fatty acids, and other metabolites.^[^ ^43^ ^]^ The AMPK lies at the crossroads of metabolically driven macrophage inflammation. The mTOR activation is maintained, within a range, by AMPK, which is known to activate mTOR during conditions of increased energy supply.^[^ ^44^ ^]^ Most studies have reported a strong association between mTOC1 and the regulation of fatty acid oxidation, further supporting the notion that the cellular metabolism of macrophages was intricately connected to their activation status.^[^ ^45^, ^46^, ^47^ ^]^

### 2.6 Extracellular ATP Modulates Macrophage Polarization via A2b Adenosine Receptor

An association study between metabolomics and genomics suggested that cAMP signaling pathway was also enriched. Pearson's correlation analysis revealed a positive association between M2 macrophages, cAMP levels, and energy metabolism (Figure 5F). This was not surprising since ATP acted as a signaling molecule in addition to being an energy source.^[^ ^9^, ^24^ ^]^ Excessive ATP was degraded into AMP and subsequently into adenosine, which may be released from the cells and appear in the extracellular space. Macrophage activation has been reported to be controlled by extracellular ATP and associated adenosine signaling.^[^ ^9^ ^]^

**Figure 6:**
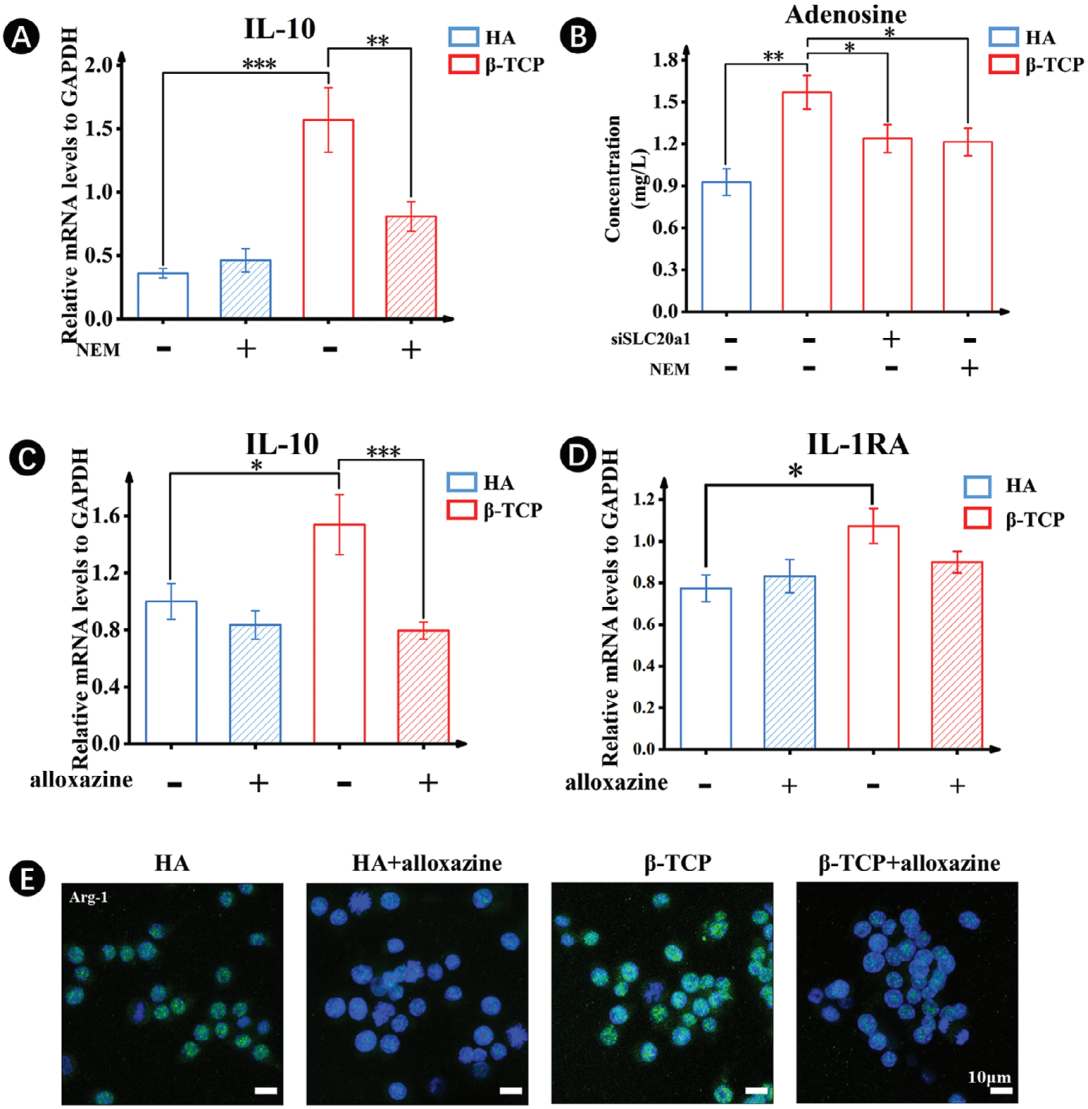
Extracellular ATP modulates macrophage polarization via A2b adenosine receptor. A) Gene expressions of IL‐10 after 5 days of culture on HA and β‐TCP scaffolds with or without vesicle transport inhibitor NEM. B) HPLC measurement of adenosine in culture medium after 5 days with or without SLC20a1 and NEM. C,D) Gene expressions of IL‐10 (C) and IL‐1RA (D) after 5 days of culture on HA and β‐TCP scaffolds with or without alloxazine. E) Immuno‐Fluorescence staining for Arg‐1. (*n* = 5. The values presented are the mean ± SD. Statistical significance is assessed by one‐way ANOVA with Tukey's post hoc test. ^*^ *p* < 0.05, ^**^ *p* < 0.01, and ^***^ *p* < 0.001).

To investigate the impact of extracellular ATP on macrophage polarization, we used the vesicular transport inhibitor N‐ethyl maleimide (NEM) to inhibit ATP transport to the extracellular environment. The results showed that the use of NEM led to the down‐regulation of IL‐10 expressions in β‐TCP scaffolds, suggesting that inhibition of ATP transport negatively influenced β‐TCP scaffold‐assisted M2 macrophage polarization (**Figure** **6**A). However, no significant inhibitory effect was observed on HA scaffolds. This might be because the inhibition of ATP transport may compensate for the intracellular energy deficiency, resulting in an increase in IL‐10 expressions. HPLC measurements revealed a substantial quantity of adenosine in cell culture involving β‐TCP scaffolds. The presence of adenosine, a metabolite of ATP, in the extracellular milieu indicated that ATP was released from cells and rapidly metabolized to adenosine through the action of membrane‐bound ectonucleotidases (CD39), including ectonucleoside triphosphate diphosphohydrolase, ectonucleotide pyrophosphatase/phosphodiesterase, and ecto‐5′nucleotidases (CD73).^[^ ^48^ ^]^ Moreover, the presence of extracellular adenosine was abolished through the SLC20a1 knockdown and the use of NEM, respectively (Figure 6B), demonstrating the correlation between PO_4_^3−^‐modulated ATP and adenosine.

To further confirm the involvement of adenosine in ATP‐mediated macrophage polarization, we investigated the effect of adenosine receptors on the modulation of macrophage polarization toward the M2 subtype. Specific pharmacological inhibition of adenosine receptors by alloxazine inhibitors demonstrated that the alloxazine down‐regulated the expressions of IL‐10 and IL‐1RA genes in the cells treated with β‐TCP scaffolds (Figure 6C,D). Immunofluorescent staining for Arg‐1 also revealed a decrease in Arg‐1 expression in the presence of alloxazine (Figure 6E). These findings aligned with the previous reports indicating the beneficial effects of adenosine signaling via the A2b adenosine receptor on macrophage polarization.^[^ ^49^, ^50^ ^]^ Studies have reported that activation of the A2b adenosine receptor generated cAMP, which can stimulate protein kinase A (PKA).^[^ ^51^ ^]^ This would be in line with our results from GeneMANIA analysis that β‐TCP scaffold increased intracellular cAMP, resulting in the polarization of macrophages toward M2, probably via PKA‐cAMP responsive element‐binding protein (CREB) signaling (Figure 5E).

**Figure 7:**
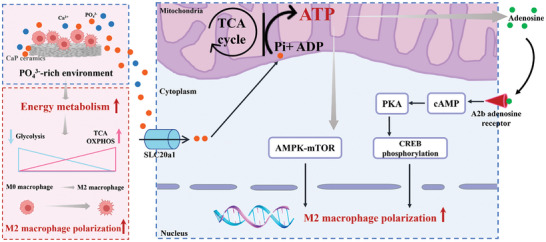
The potential molecular mechanism behind the enhanced M2 macrophage polarization of phosphate ions released from CaP ceramics.

The findings collectively suggested a molecular mechanism, as illustrated in **Figure** **7**, wherein the fluctuating dissolution and precipitation of CaP minerals from HA and β‐TCP ceramics regulated the levels of calcium ions (Ca^2+^) and phosphate ions (PO_4_^3−^) in the extracellular environment. The extracellular PO_4_^3−^ was transported into macrophages via the SLC20a1 transporter and subsequently entered the intracellular compartment. High levels of intracellular PO_4_^3−^ drove the TCA cycle, followed by oxidative phosphorylation, resulting in rapid and efficient energy production. Importantly, our study indicated that the promotion of oxidative phosphorylation was a promising strategy for modulating macrophage polarization. In contrast to M1 macrophages, M2 macrophages preferred oxidative phosphorylation as a means of storing energy and supporting metabolism. Alterations in metabolic pathways played a crucial role in determining the distinct macrophage polarization phenotypes. Additionally, macrophage polarization led to metabolic reprogramming, with macrophages selectively utilizing metabolic pathways to enhance the activation of phenotypes and effector functions.

The positive effects of TCA cycle and OXPHOS on macrophage polarization were not only limited to supporting bioenergetics and biosynthesis but also altered transcription by modulation of epigenetic modifications leading to changes in transcriptional programs. The potential molecular mechanism behind the enhanced M2 macrophage polarization of phosphate ions released from CaP ceramic was attributed to the following two points. On the one hand, the active function of PO_4_^3−^ in macrophage polarization involved AMPK‐mTOR axis, likely through the increased energy metabolism. AMPK‐mTOR axis acted as central metabolic switch in cell metabolism and served as a signaling molecule in macrophages. When cell energy was increased, there was a decrease in the activation of AMPK, resulting in turning on the mTOR signaling cascade. On the other hand, intracellular ATP could be secreted into the exocellular space and metabolized into adenosine, which subsequently promoted the polarization of macrophages toward an M2 phenotype through the A2b adenosine receptor via autocrine and/or paracrine signaling.

Notably, although this study primarily focused on phosphate metabolism in a mineralized environment, it did not discount the positive effects of Ca moieties found in CaP ceramics. Previous research has already elucidated the molecular mechanism through which calcium influenced M2 macrophage polarization via PI3K‐Akt and Wnt/β‐catenin.^[^ ^52^ ^]^ Moreover, the CaP moieties of the CaP ceramics usually underwent dissolution/precipitation responding to the concentration of Ca^2+^ or PO_4_^3‐^ ions in the surrounding environment, resulting in the creation of a dynamic environment. This dynamic mineral environment held significant importance, as it demonstrated the interdependency between extracellular Ca^2+^, PO_4_^3−^, and Ca.

In summary, we have gained insights into the impact of phosphate ions on the progression of polarization, specifically highlighting the crucial expression pattern of the SLC20a1 phosphate transporter during macrophage polarization and elucidating a mechanism that emphasized the metabolic regulation of phosphate ions to enhance cellular energy status for M2 phenotype polarization. These findings not only established phosphate ion as a favorable metabolic factor for macrophage polarization toward the M2 phenotype but also underscored the importance of considering the regulation of PO_4_^3−^ content in the design of CaP‐biomaterials.

## 3 Conclusion

Here, we report the molecular mechanism by which phosphate ions promote macrophage polarization toward the M2 phenotype via metabolic regulation. In vitro and in vivo studies demonstrate that extracellular phosphate ions enter the cells through SLC20a1 and generate large amounts of ATP through the TCA cycle and OXPHOS. Furthermore, the integration of metabolome and transcriptome analysis has established a link between phosphate‐mediated macrophage polarization, metabolism, and intracellular signal transduction. Activation of the intracellular AMPK‐mTOR axis has been identified as a contributing factor to M2‐macrophage polarization, potentially due to the enhanced energy supply from increased phosphate ion levels. In addition, extracellular phosphate regulates the synthesis of ATP synthesis, which is subsequently secreted and metabolized to adenosine, thereby promoting M2 macrophage polarization via A2b adenosine receptors. This research significantly contributes to our understanding of how phosphate governs macrophage phenotype through metabolic processes and provides ideas for the design of CaP ceramic materials in subsequent bone repair.

## 4 Experimental Section

### Characterization of HA and β‐TCP Scaffolds

Engineering Research Center in Biomaterials, Sichuan University provided porous HA and β‐TCP scaffolds in two different sizes (Ф14 × 2 mm for cell culture and Ф2 × 6 mm for animal experiment). The microtopography and elemental composition of the CaP ceramics were observed using field emission scanning electron microscopy (FE‐SEM, S‐4800, Hitachi, Japan) and EDS (Phenom, Philips, Netherlands). XRD (X'Pert 1, Philips, Netherlands) was performed to analyze the phase composition of CaP ceramics. To evaluate the degradation of HA and β‐TCP ceramics, samples were immersed in Tris‐HCl (0.1 mol L^−1^, PH 7.4) at 37 °C. After 1, 3, 5, and 7 days of incubation, the immersion solution (0.5 mL) was collected and added to a new 0.5 mL Tris‐HCl solution. The concentrations of PO_4_^3−^ ions were then measured using an inductively coupled plasma mass optical emission spectrometer (ICP‐OES, SPECTRO ARCOS, Spectro Analytical, Germany). To evaluate the microtopography after the degradation of the CaP ceramics, they were rinsed with deionized water, dried, and examined by SEM after 7 days of incubation.

### Cell Culture and Media

The RAW 264.7 macrophages from mice were cultured in Dulbecco's modified Eagle's medium (DMEM, Gibco, USA) supplemented with 10% fetal bovine serum (FBS, BI, ISR) and 1% penicillin/streptomycin (HyClone, USA) and cultured with a fully humidified atmosphere of 5% CO_2_ at 37 °C. The phosphorus‐rich medium was prepared as follows. To obtain final concentrations of 1 and 5 mm PO_4_^3−^ ions, various concentrations of PO_4_^3−^, NaH_2_PO_4_ and Na_2_HPO_4_ were mixed at a ratio of 1:4 to supplement the growth medium.

### Flow Cytometry

RAW264.7 (1 × 10^4^) cells cultured with HA and β‐TCP samples for 2 and 5 days were collected and then respectively incubated with peridinin chlorophyll protein (Percp, BioLegend, USA)‐conjugated anti‐mouse CD11c and activated protein C (APC, eBioscience, USA)‐conjugated anti‐mouse CD206 at room temperature for 30 min without light after fixation, membrane breaking and blocked with CD16/32 (BD pharmingen) steps. Finally, the cells were resuspended in 200 µL PBS and subjected to FACSCalibur flow cytometry (Beckman, CytoFLEXS, USA).

### Quantitative Real‐Time PCR Analysis

Total RNA was extracted and cDNA was generated using Triozl reagent and the Evo MMLVRT Mix kit (Accurate Biotechnology (Hunan) Co., Ltd), respectively, following the manufacturer's protocol. The qRT‐PCR was conducted at 95 °C (30 s), followed by 40 cycles at 95 °C (5 s)/60 °C (30 s). Signals were detected using SYBR green intercalating dye (Accurate Biotechnology (Hunan) Co., Ltd). Sequences of the primers used in this study were provided in (Table S9, Supporting Information).

### Immunofluorescence Staining

For immunofluorescence staining, RAW264.7 macrophages plated on the materials for 5 days were washed, fixed, and permeabilized with 4% paraformaldehyde (0.1% Triton X‐100). After washing with PBS, blocking was performed by 30‐min incubation with 1% bovine serum albumin (BSA). Next, the cells were incubated overnight at 4 °C with primary antibody against Arg‐1 (1:200; Abclonal, China). On day 2, the cells were incubated at room temperature with a secondary antibody conjugated to Alexa Fluor 488 for 1 h. Finally, cell nuclei were stained with DAPI for 5 min.

### Measurement of SLC20a1 Expression

For the examination of SLC20a1 expression, total RNAs from the cells treated with HA, β‐TCP, phosphorus‐rich medium (1 mm), and phosphorus‐rich medium (5 mm) were extracted with Trizol reagent and the relative expressions of SLC20a1 gene were determined using qRT‐PCR following the method mentioned above. Moreover, immunofluorescence staining was used to determine the expression levels of SLC20a1 protein.

### siRNA Knockdown

As directed by the manufacturer, RAW264.7 cells were transfected with siRNA oligonucleotides to knockdown SLC20a1. Briefly, RAW264.7 cells were transfected under serum‐free conditions with 30 nm siRNA targeting SLC20a1 and scrambled control siRNA (medium GC content) using the RNAimax transfection reagent (Invitrogen). The siRNA sequences of siRNA were given in (Table S10, Supporting Information).

### Effect of Knockdown of SLC20a1 on Macrophage Polarization

After knocking down SLC20a1, the expressions of IL‐10 and IL‐1RA were assessed by quantitative qRT‐PCR. Besides, Arg‐1 was stained by incubation with an Arg‐1 antibody (1:200; Abclonal, China) to observe its expression of Arg‐1 in vitro.

### Inorganic Phosphate Assay

The effects of HA and β‐TCP ceramics on intracellular phosphate contents were measured by malachite green phosphate detection kit. In brief, RAW264.7 was seeded on HA and β‐TCP ceramics in 24‐well culture plates. On day 5, cells were rinsed three times with physiological saline solution before adding deionized water (200 µL per well). The cells were frozen and thawed three times to lyse them. Subsequently, the assay was performed in accordance with the manufacturer's instructions (Beyotime).

### Luminescent ATP Analysis and Fluorescent ATP Staining

The intracellular ATP concentration was quantified using an ATP assay kit (Beyotime). In brief, after counting the cells, they were completely lysed by adding lysate. Then, the supernatants were harvested by centrifugation at 4 °C, 12 000 g for 5 min. Afterward, the ATP content of individual cell was determined according to the manufacturer's instructions.

For subsequent fluorescent staining of intracellular ATP, the cells were gently rinsed with PBS three times and then were incubated in serum‐free DMEM dissolved in 30 µm quinacrine dihydrochloride (Sigma–Aldrich) for 30 min before imaging with an inverted fluorescence microscope (Leica, DMi8, Germany).

### Detection of Targeted Energy Metabolites

After 5 days of HA and β‐TCP treatment respectively, RAW264.7 cells were collected and centrifuged at 1200 r min^−1^ for 5 min at 4 °C, before washing them in cold PBS. Further centrifugation at 1200 r min^−1^ for 5 min at 4 °C was followed by vigorous vortexing with 1 mL of 80% cold methanol. The supernatant was collected and normalized to the protein concentration. Subsequently, the sample extracts were analyzed using an LC‐ESI‐MS/MS system (Waters ACQUITY H‐Class; MS, QTRAP 6500+ System). The AB 6500+ QTRAP LC‐MS/MS system was equipped with an ESI Turbo Ion‐Spray interface and can operate in both positive and negative ion modes according to Analyst 1.6 software (A B Sciex). Detection and determination of the relative abundances of the targeted energy metabolites were performed using MetWare based on the AB Sciex QTRAP 655 LC‐MS/MS platform.

### Observed Metabolite Alterations Using Untargeted Metabolomics

The preparation and collection of samples, and untargeted metabolomics analysis were performed as described previously. In short, intracellular metabolites were analyzed using an LC‐ESI‐MS/MS system (UPLC, ExionLC AD; MS, QTRAP System). LIT and triple quadrupole (QQQ) scans were acquired using a triple quadrupole‐linear ion trap mass spectrometer (QTRAP), QTRAP LC‐MS/MS System which was equipped with an ESI Turbo Ion‐Spary interface and could operate in positive and negative ion mode by Analyst 1.6.3 software (Sciex).

### RNA‐Sequencing and Data Analysis

RNA‐sequence (RNA‐seq) analysis was performed as described previously. Total RNA isolated from macrophages was selected for RNA sequencing. The KEGG pathway and GO analysis were used for the enrichment analysis of differentially expressed genes. Genes with differential expression were identified by calculating the fold‐change over two and an adjusted P ‐value of 0.05.

### Verification of the AMPK‐mTOR Axis

After 5 days of culture on HA and β‐TCP scaffolds, macrophages were stained to visualize AMPK (1:200; Abclonal) and mTOR (1:200; Abclonal) using immunofluorescence staining as previously described. Additionally, qRT‐PCR was used to detect the mRNA expression levels of mTOR as previously described. To further confirm that phosphate ions were involved in AMPK‐mTOR axis, SLC20a1 expression was knocked down. After 5 days, immunofluorescence staining was used to explore the expressions of AMPK and mTOR. In addition, mTOR gene expression levels were determined using qRT‐PCR. Finally, Torin 1 (a specific mTOR inhibitor, sigma) was used at a concentration of 250 nm to block the mTOR activity. Afterward, IL‐10 expressions were measured via qRT‐PCR.

### Adenosine Content

Prior to the experiments, the cell culture media (1 mL) was lyophilized for 24 h and frozen at −80 °C. Using HPLC‐UV, the concentration of adenosine was quantified. Specifically, chromatic separations were conducted on an Aeris Peptide 3.6u Xb‐C18 phenomenex column (150 mm × 2.10 mm) with the assistance of a Hitachi Elite LaChrom L‐2130 pump equipped with a UV–vis detector (Hitachi Elite LaChromL‐2420). Adenosine levels were detected at 254 nm under isocratic conditions, with a flow rate of 0.23 mL·min^−1^ and an injection volume of 20 µL. The mobile phase for adenosine consisted of 0.5% acetonitrile, 5% (vol/vol) methanol, and 94.5% (vol/vol) sodium acetate buffer (0.25 m, pH 6.3).

### Blockade of ATP Transport

NEM was added to the culture medium (final concentration, 0.5 µm) to inhibit ATP transport, and the fresh medium was changed after 2 days. Subsequently, the expression of IL‐10 was determined using qRT‐PCR as described above.

### Alloxazine Inhibit the A2b Adenosine Receptor Expression

The A2b receptor inhibitor alloxazine (Sigma–Aldrich) was added at a concentration of 100 nm to the medium for pharmacological inhibition of adenosine receptors. After 2 days, culture medium was replaced. Next, the expressions of IL‐10 and IL‐1RA genes were determined using qRT‐PCR following the method mentioned above. Moreover, immunofluorescence staining was used to determine the expression level of Arg‐1 protein.

### In Vivo Evaluation

The animal in vivo experiments were performed in accordance with the Institutional Animal Care and Use Committee of Sichuan University (Chengdu, China) guidelines (approval No. K2023011). Male Sprague‐Dawley rats (weighing 350–400 g), purchased from DaShuo Experimental Animal Co. Ltd. (Chengdu, China), were used in this study. The rats were anesthetized by intraperitoneal injection of chloral hydrate at a dosage of 0.4 mL/100 g, and dental trephine bars were used to create 2 mm in diameter and 4.5 mm in‐depth critical‐sized femur defects. After washing the holes with sterile saline, the implants were inserted into the defects. After all the surgical procedures, the rats were housed in standard cages and fed a regular laboratory diet.

The rats were sacrificed after 7 and 14 days of implantation, and the tibias with implants were harvested for subsequent analysis. The hematoxylin & eosin (H&E) staining and immunofluorescence staining for CD163 (NOVUS, 1:100), CD 86 (HUAAN, 1:200), and SLC20a1 (BIOSS, 1:100) were used to detect macrophage polarization and new bone formation.

### Statistical Analysis

All data were present as mean ± standard deviation (SD) from at least three repeated experiments. Student's *t*‐test was used to determine the significance between the two groups. One‐way analysis of variance (ANOVA) followed by Tukey's post hoc test was utilized to perform statistical comparisons between multiple groups. The detailed sample sizes were labeled in figure caption, and the sample size for each statistical analysis (n) was at least three. GraphPad Prism 8.00 (GraphPad Software Inc., USA) was utilized for data analysis. The significance levels were denoted by asterisks: ^*^ *p* < 0.05, ^**^ *p* < 0.01, and ^***^ *p* < 0.001, respectively. A value of *p* < 0.05 was considered indicative of a statistically significant difference.

## Conflict of Interest

The authors declare no conflict of interest.

## Supporting information

Supporting Information

## Data Availability

The data that support the findings of this study are available from the corresponding author upon reasonable request.
