# Supplementary material for: Inorganic Phosphate as “Bioenergetic Messenger” Triggers M2‐Type Macrophage Polarization
Source: Adv Sci (Weinh). 2024 Jan 21;11(13):2306062. doi: 10.1002/advs.202306062 (PMC10987138; doi:10.1002/advs.202306062)
Supplement: Supplementary file 1 — Supporting Information [file ADVS-11-2306062-s001.pdf]

ADVANCED SCIENCE
Open Access

Supporting Information

for *Adv. Sci.*, DOI 10.1002/advs.202306062

Inorganic Phosphate as "Bioenergetic Messenger" Triggers M2-Type Macrophage Polarization

Xiaoqing Sun, Zhiyu Li, Xiang Wang, Jing He and Yao Wu**

Supporting Information

Inorganic phosphate as “bioenergetic messenger” triggers M2-type macrophage polarization

Xiaoqing Sun, Zhiyu Li, Xiang Wang, Jing He, Yao Wu**

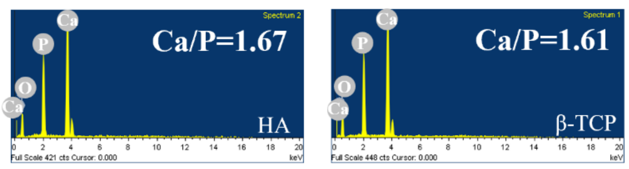

Figure S1. EDS spectrum of HA and β-TCP.

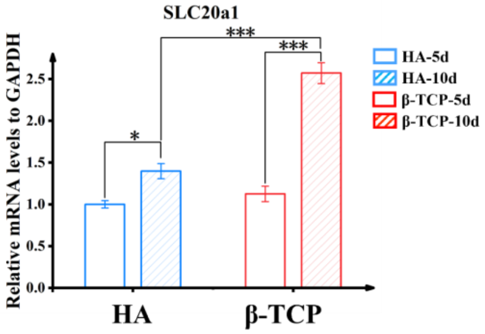

Figure S2. Gene expressions of SLC20a1 after 5 and 10 days of culture on HA and β-TCP scaffolds.

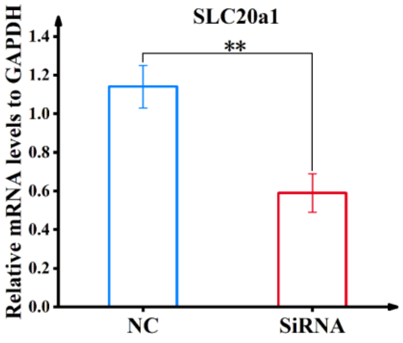

Figure S3. RT-qPCR analysis of SLC20a1 gene of RAW264.7 cells of siRNA transfection.

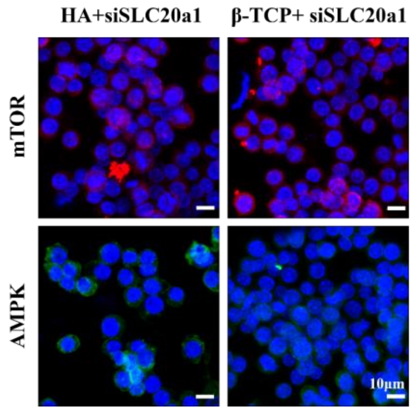

Figure S4. Immuno-Fluorescence staining for mTOR and AMPK after 5 days of culture on HA and β-TCP scaffolds with SLC20a1 siRNA.

Table S1. The content of metabolites about HA and β-TCP.

**Sample**	**HA**	**β-TCP**
3-phenyllactic-acid	0.4284	0.6082
Lactate	306.3230	611.9177
Alpha-Ketoglutaric-Acid	35.2434	59.3673
Succinic Acid	20.5891	30.8705
Fumaric-acid	18.5921	27.9945
Citric-acid	9.4233	16.0284

Table S2. List of the genes significantly changed at both the transcriptome and metabolome levels related to the HA and β-TCP (FPKM value).

**Gene**	**HA**	**β-TCP**
Tnnt2	0.0709	3.7909
Gm14275	0	28.1093
Cth	1.4958	16.2145
Syn1	0.3996	6.0948
Naa80	0	1.7073
Dnmt3l	0.0552	2.0513
Lst1	37.9895	163.433
Serpinb7	0	2.2539

Aqp9	0.2519	2.0325
Klra2	1.0419	6.1268
Ubald2	16.9961	57.0226
Iba57	0.2024	1.5479
Zyx	4.311	14.1882
Ccl4	84.3132	258.8212
Kctd11	0	0.8922
Nfkbil1	2.8316	9.8755
Gm11707	0	5.027
Pkn3	3.1243	8.9259
Alkbh6	0.8619	3.3567
Plekhj1	10.3815	27.5629
Pafah2	2.6281	7.4299
Zswim7	2.4765	10.5647
Fas	1.2787	4.7783
Icam5	0	0.7192
Glrp1	0.7298	3.3662
Grap	14.5527	36.4217
Msi1	0.3788	1.4519
1110002L01Rik	0.359	1.5947
Acta2	0.2251	1.6956
Ftl1-ps1	10.8248	27.7757
Msrb1	16.5496	39.5708
Bad	3.028	8.0619
Sqstm1	56.0129	123.9227
Gpx1	161.7862	355.7514
Atf4	73.5696	161.7469
Aplp1	0.4916	1.9747
Egr2	35.1452	13.8406
Cpne2	2.9526	7.3754
Il20rb	0	0.4086
Pkmyt1	4.7951	11.2273
Fam174c	1.1343	5.9988
Isca2	3.0186	8.1033

Cnrip1	0.2607	1.1591
Ddit3	2.2127	6.2869
Emc6	28.6892	61.8995
Cdk20	0.4378	1.319
Fam221b	2.0415	4.8808
Nenf	13.7702	32.3479
Pla2g2e	0	1.6242
Tctn2	0	0.179
Eno2	7.0519	2.7516
Xlr	5.6981	12.8172
Dap	70.1425	141.113
Soat2	0.7199	2.4591
Mrpl54	15.9804	35.065
Hoxa4	0	0.8625
4930579G18Rik	0	1.4203
Selenoh	44.1501	89.3454
Cox6b1	63.6225	128.8898
Tmem222	10.7232	22.1358
Atp5mpl	41.0753	81.196
Glrx	11.3285	22.188
4930430E12Rik	1.3552	4.4919
Mrpl57	9.3103	18.7589
Plk3	8.3994	17.0427
Slc25a43	1.4813	4.055
Slc6a13	3.0266	6.549
Acot8	2.8058	6.4542
Rps28	28.2812	54.4518
Bhlhe41	8.5342	3.836
Ceacam19	0.1961	0.8863
Adm	8.8218	3.4321
Dipk1b	0	0.4627
Ss18l1	1.7834	0.5936
Slfn2	19.9065	37.9715
Ifi27l2a	36.5426	14.9706

Smim3	1.3999	2.8693
Igfbp7	0	0.9003
Pfdn1	8.9656	18.2855
Tmco4	2.3678	4.9064
Mrpl42	7.6088	14.3755
Fzd7	6.606	2.9451
Rhbdd3	2.4611	5.1741
Lsp1	6.2959	2.8786
Cpxm1	0	0.424
Cdyl2	1.6177	3.7303
Entpd1	1.4964	0.5749
Gm20633	0.0194	0.4047
Lsm10	4.136	8.7476
Tmem80	1.1581	2.4334
Lpar6	1.6175	0.6789
Il12rb1	0.174	0.5861
Zfp870	1.7522	0.7666
2310009B15Rik	2.6995	5.3029
Vmn1r88	0.2771	0.6094
Dgkg	1.0684	0.489
Gask1b	0.1145	0.3044
Rnf26	0.6488	1.3216
Xkr5	0.7031	0.249
Pkd1l2	0.3904	0.7384
Pard6g	0.768	0.2548
Rtp4	2.1911	0.9677
Camk2n2	1.9573	3.7273
Bcdin3d	0.3543	0.7265
Acot1	0.6728	0.2777
Spata1	0.2132	0.4863
Zfp677	0.9416	0.4124
Rpl29	3.1711	1.4754
Dnai4	0.4285	0.1882
Prss36	0.2388	0.5513

D330041H03Rik	0.8499	0.3973
Gm44950	0.8374	0.3243
Gm49396	0.9512	0.4428
Plxdc1	0.4657	0.2014
Fv1	1.8709	0.8717
Hoxa6	1.9484	0.7865

Table S3. List of the genes' expression (Log2fold change) about AMPK signaling pathway.

**Gene**	**Log$_2$fold change**
Igf1	-3.85367
Irs3	-2.27177
Ppp2r3a	-1.09516
Sirt1	-1.28199

Table S4. List of the genes' expression (Log2Fold change) about energy metabolism.

**Gene**	**Log$_2$fold change**
Ass1	-1.8437
Uap1	-1.60728
Cmah	-5.44284
Hpgds	-1.51902
Pde8b	-1.01783
Xdh	-1.08624
Tomt	-2.57287
Ugt1a6a	-2.74303
Rgn	-7.45675
Me2	-1.02382

Table S5. List of the genes' expression (Log2Fold change) about mTOR signaling pathway.

**Gene**	**Log$_2$fold change**
Lpin3	8.754937
Fzd7	-1.07132
Seh1l	-1.02302
Deptor	-1.53907

Table S6. List of the genes' expression (Log2Fold change) about cAMP signaling pathway.

Gene	Log_2fold change
Rap1a	-1.44056
Pde4b	-1.31705
Adcy6	-3.93491
Adrb2	-1.46658
Atp2b3	-7.68686
Grin1	-7.83816
Htr1b	-8.10014
Ppp1cb	-1.18284

Table S7. List of the genes' expression (Log2Fold change) about PI3K-Akt signaling pathway.

Gene	Log_2fold change
Col4a6	-1.14515
Fgf2	-8.88221
Itga4	-1.85363
Itgav	-1.16698
Itgb1	-1.08567
Lama4	-1.23199
Sgk3	-1.50614
Pgf	-2.53641

Table S8. List of the genes' expression (Log2Fold change) about M2 macrophage.

Gene	Log_2fold change
Amd1	-1.14476
Amd2	-1.34448
Nos1	-1.97456
Ass1	-1.8437
Glul	-1.18117
Gls2	-1.48738

Table S9. Primer sequences of each gene.

Target	Forward	Reverse
GAPDH	TGTGTCCGTCGTGGATCTGA	TTGCTGTTGAAGTCGCAGGAG
IL-10	GCCAGAGCCACATGCTCCTA	GATAAGGCTTGGCAACCCAAGTAA
IL-1RA	ACCAGGGCATCACATATACTAAGC	AAACCTCTTGCCGACATGGAA

CD206	AGGATCATACTTCCCTGCTTGCTA	CACCCTCCATCTATCCGTCCAA
SLC20a1	TCTGGGCTCATGTGACTTGC	GGCGGTCACAAGCATGAAAA

Table S10. Sequences of siRNA targeting SLC20a1.

sense	antisense
GGGUGUCAAGUGGUCUGAACUGAUA	UAUCAGUUCAGACCACUUGACACCC
